# Effects of C8 nerve root block during interscalene brachial plexus block on anesthesia of the posterior shoulder in patients undergoing arthroscopic shoulder surgery: study protocol for a prospective randomized parallel-group controlled trial

**DOI:** 10.1186/s13063-019-3624-9

**Published:** 2019-08-28

**Authors:** Eugene Kim, Chang Hyuk Choi, Jong Hae Kim

**Affiliations:** 1 Department of Anesthesiology and Pain Medicine, Hanyang University Medical Center, College of Medicine, Hanyang University, Seoul, Republic of Korea; 2 Department of Orthopaedic Surgery, School of Medicine, Daegu Catholic University, Daegu, Republic of Korea; 3 Department of Anesthesiology and Pain Medicine, Daegu Catholic University Medical Center, School of Medicine, Daegu Catholic University, 33, Duryugongwon-ro 17 gil, Nam-gu, Daegu, 42472 Republic of Korea

**Keywords:** Interscalene brachial plexus block, Cervical nerve root, Arthroscopic shoulder surgery, Lower trunk of the brachial plexus

## Abstract

**Background:**

A classical approach to produce interscalene brachial plexus block (ISBPB) consistently spares the posterior aspect of the shoulder and ulnar sides of the elbow, forearm, and hand, which are innervated by the lower trunk of the brachial plexus (C8–T1). As an alternative to the classical approach, a caudal approach to ISBPB successfully produces anesthesia of the ulnar sides of the elbow, forearm, and hand. However, its beneficial effects on anesthesia in the posterior aspect of the shoulder have not been investigated. In addition, the C8 nerve root is not routinely selectively blocked during ISBPB. Therefore, we will compare the C5 to C7 and C5 to C8 nerve root blocks during a caudal approach to ISBPB to assess the clinical benefit of C8 nerve blocks for the surgical anesthesia of the posterior aspect of the shoulder.

**Methods/design:**

In this prospective parallel-group single-blind randomized controlled trial, 74 patients scheduled to undergo arthroscopic shoulder surgery under ISBPB are randomly allocated to receive the C5 to C7 or C5 to C8 nerve root block at a 1:1 ratio. The primary outcome is pain intensity, which is rated as 0 (no pain), 1 (mild pain), or 2 (severe pain), during the introduction of a posterior portal into the glenohumeral joint. The secondary outcomes are (1) the extent of the ipsilateral sensory, motor, hemidiaphragmatic, and stellate ganglion blockade, (2) changes in the results of a pulmonary function test, (3) incidence of complications related to ISBPB, (4) postoperative numerical pain rating scale scores, (5) patients’ satisfaction with the ISBPB, (6) dose and frequency of analgesic use, and (7) incidence of conversion to general anesthesia.

**Discussion:**

This study is the first to evaluate the beneficial effects of the C8 nerve root block during ISBPB, which has rarely been performed due to the technical challenge in visualizing and blocking the C8 nerve root. It is expected that a C8 nerve root block performed during ISBPB will provide sufficient surgical anesthesia of the posterior aspect of the shoulder, which cannot be achieved by a classical approach to ISBPB.

**Trial registration:**

ClicnicalTrials.gov, NCT03487874. Registered on 4 April 2018.

**Electronic supplementary material:**

The online version of this article (10.1186/s13063-019-3624-9) contains supplementary material, which is available to authorized users.

## Background

Since Winnie introduced the interscalene brachial plexus block (ISBPB) in 1970 [1], it has been widely used for surgical anesthesia [2], as a supplement to general anesthesia [3], and for postoperative analgesia [4] in patients undergoing arthroscopic shoulder surgery. However, a conventional ISBPB is performed at the C6 level [5], and the lower trunk of the brachial plexus (C8–T1) is spared [6]. For that reason, patients undergoing conventional ISBPB complain of pain or discomfort in the posterior aspect of their shoulder [7].

To block the lower trunk of the brachial plexus, a caudal approach to ISBPB guided by nerve stimulation [8, 9], ultrasound [10], or both [11] can be performed. Although it can produce sufficient anesthesia in the ulnar sides of the elbow, forearm, and hand, which are innervated by the lower trunk, [8–11], whether the caudal approach to ISBPB provides anesthesia in the posterior aspect of the shoulder into which a posterior portal is introduced for the examination of the glenohumeral joint during arthroscopic shoulder surgery has not been evaluated. Furthermore, the use of nerve stimulation for a surface landmark technique [8, 9] does not guarantee the correct placement of a local anesthetic around each nerve root or trunk of the brachial plexus [12]. Even under ultrasound guidance, which directly shows the spread of the local anesthetic around nerves [13], the lower trunk of the brachial plexus cannot easily be visualized because the C8 and T1 nerve roots are posterior to the subclavian artery, which means they are located more deeply [13–15]. Fortunately, the C8 nerve root, which is located less deeply than the T1 nerve root, was found to be observable in 80% of subjects under ultrasound guidance [16]. However, to date, no significant attempts have been made to block the C8 nerve root accurately or even the other cervical nerve roots [10, 11].

Therefore, we will compare pain intensity upon the introduction of a posterior portal into the subacromial space between patients receiving the C5 to C7 nerve root block and those receiving the C5 to C8 nerve root block during a caudal approach to ISBPB. Our primary hypothesis is that the C5 to C8 nerve root block will reduce the intensity of pain caused by the introduction of a posterior portal compared to the C5 to C7 nerve root block.

## Methods/design

### Study design

**Fig. 1:**
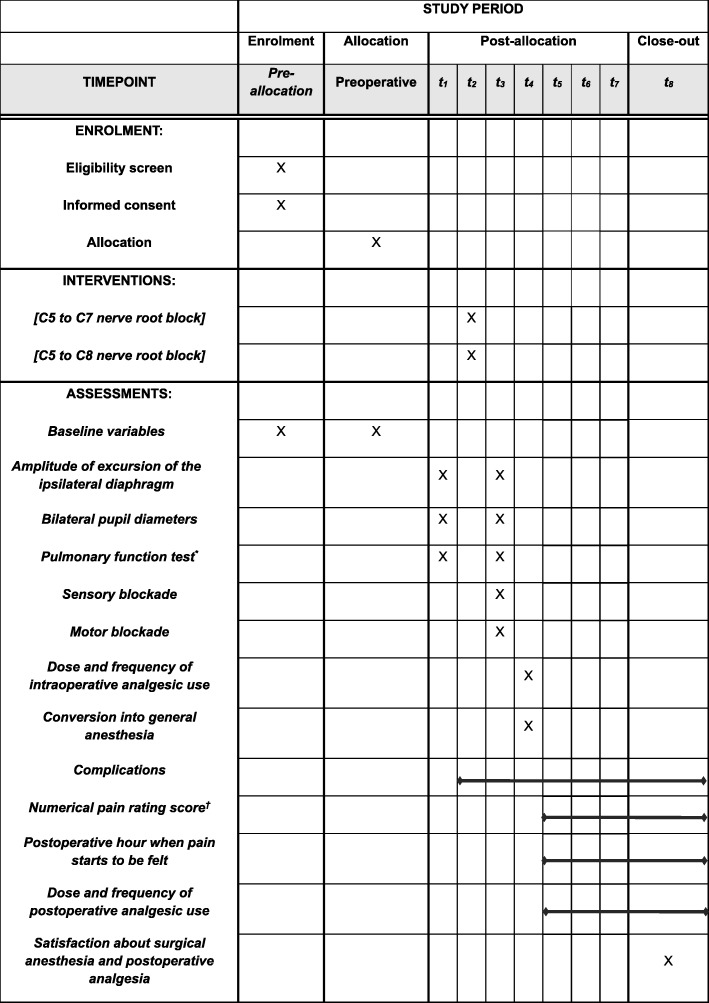
The SPIRIT flow diagram: the schedule of enrolment, interventions, and assessments. t_1_ is before the placement of the interscalene brachial plexus block. t_2_ is when the interscalene brachial plexus block is placed. t_3_ is 30 min after the placement of the interscalene brachial plexus block. t_4_ is during surgery. t_5_ is on admission to the postanesthetic care unit. t_6_ is on discharge from the postanesthetic care unit. t_7_ is between 6 and 12 h after surgery. t_8_ is 24 h after surgery. * Forced expiratory volume at 1 s (FEV1) and FEV1/forced vital capacity. † Including worst numerical pain rating score.

The protocol of this prospective single-center parallel-group single-blind randomized controlled trial was approved by the institutional review board of Daegu Catholic University Medical Center (CR-18-018) and was registered in ClinicalTrials.gov (NCT03487874) before patients were enrolled. This trial is being conducted in a tertiary university hospital (Daegu Catholic University Medical Center) in Daegu, Republic of Korea. The protocol of this trial conforms to the Standard Protocol Items: Recommendations for Interventional Trials (SPIRIT) guidelines (Fig. 1 and Additional file 1). The schedule of enrollment, intervention, and assessments is based on the SPIRIT diagram. The final report of this trial will be written in accordance with the Consolidated Standards of Reporting Trials (CONSORT) statement.

### Participants

Patients aged between 20 and 80 years with an American Society of Anesthesiologists physical status of 1 or 2 and who are scheduled to undergo arthroscopic shoulder surgery under ISBPB will be enrolled in this trial. Candidate participants who fulfill the inclusion criteria will be recruited during a visit to the outpatient department or during the preoperative visit the day before surgery. The following are the exclusion criteria:

- Patient refusal
- Contralateral hemidiaphragmatic paralysis or paresis
- Contralateral vocal cord palsy
- Severe pulmonary restrictive disease
- Coagulopathy
- Allergy to local anesthetics or history of allergic shock
- Difficulty communicating with medical personnel
- Peripheral neuropathy or neurologic sequelae on the operative limb

### Ethics, consent, and permissions

Written informed consents will be obtained from all participants by JHK before they are enrolled in the study.

### Randomization and blinding

**Fig. 2:**
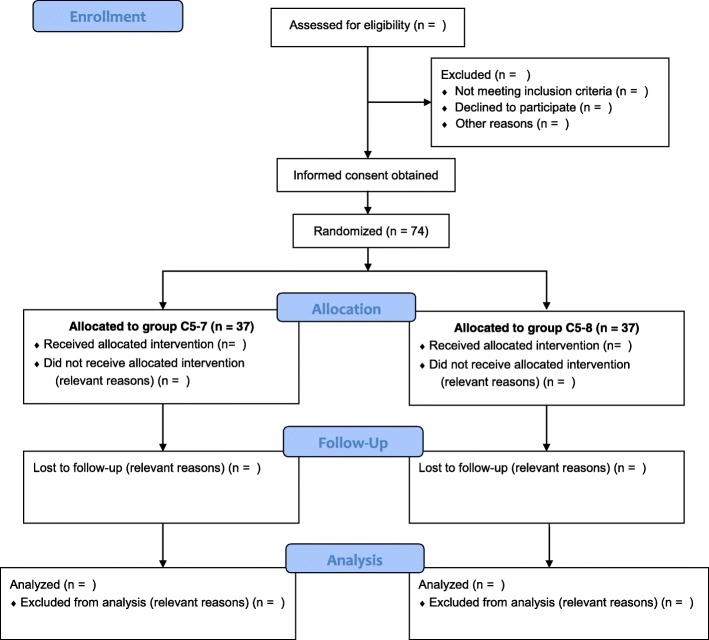
CONSORT flow chart

Following recruitment, the participants will be randomly allocated to one of the two groups: (1) those receiving the C5 to C7 nerve root block during ISBPB and (2) those receiving the C5 to C8 nerve root block. They will be allocated in a 1:1 ratio (Fig. 2) following block randomization with randomly selected block sizes of 2, 4, and 6 using random numbers generated by Microsoft Excel 2016 (Microsoft Corp., Redmond, WA, United States) [17]. The random sequence will be generated and managed by an assistant who is not involved with this study. The assistant will place each randomization code within a sealed opaque envelope. Once a patient is enrolled in this study, an anesthesiologist will open the sealed opaque envelope and will perform the allocated procedure.

Allocations will be blinded to all participants and outcome assessors except for the anesthesiologist (JHK) performing ISBPB. The allocation will not be revealed accidentally to the participant because the block needle is introduced through the same area for both types of ISBPB. Moreover, the ultrasound monitor (ProSound α7 Premier, Hitachi Aloka Medical, Ltd., Tokyo, Japan) is not within the participant’s line of sight and the participant’s head will be covered with a sterilized drape. If serious adverse events that significantly affect the safety of participants occur (e.g., cardiovascular collapse, apnea, coma, or seizure due to local anesthetic systemic toxicity), the patients will be removed from the trial, the blinding will be removed, and the events will be reported to the institutional review board.

### Withdrawal, dropout, and discontinuation

Participants may withdraw from the study at any time. The investigators can also withdraw a participant if it is considered that their safety will be compromised by their continued participation. In addition, a participant will be withdrawn if unexpected changes in their surgical plan necessitate general anesthesia. The reasons for withdrawal will be recorded in the case report form.

### Confidentiality

Participants’ private information, such as their name, social security number, phone number, address, family relationship, educational background, occupation, or chart number, will not be collected. Only the study code will be collected, which will be managed separately. The data collected will be kept confidential until they are required for analysis by the investigators. The principal investigator (JHK) will manage the final dataset. The data collected will be stored under encryption for 3 years after the completion of the study and subsequently discarded.

### Intervention

Patients fast from midnight before the day of surgery. The infusion of Plasmalyte is commenced at a rate of 5 ml kg^-1^ hr^-1^ via a peripheral intravenous line 1 hr before surgery. No premedication is administered to the patients. On arrival at the operating theater, electrocardiography, pulse oximetry, and noninvasive blood pressure monitoring are instituted. To facilitate the placement of ISBPB, patients’ heads are slightly rotated to the contralateral side to the block and their necks are extended in a supine position. The skin around the neck and clavicle is sterilized using povidone iodine and is then covered with a fenestrated drape.

**Fig. 3:**
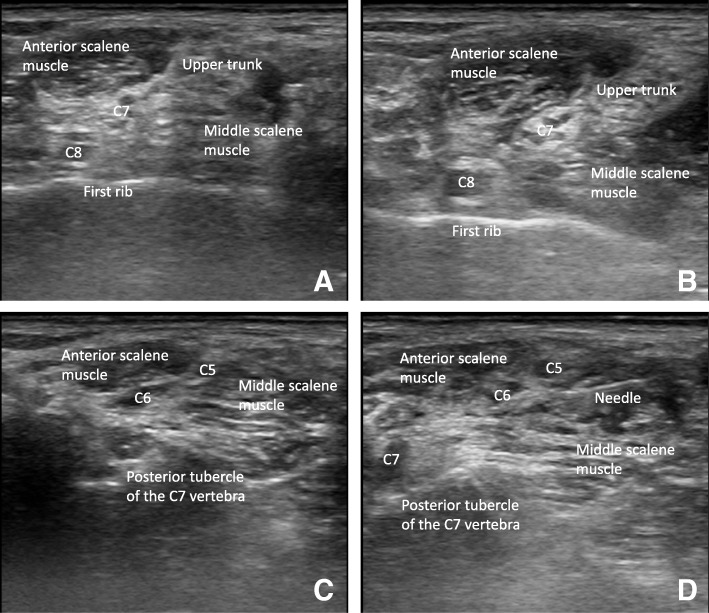
Ultrasound-guided C5 to C8 nerve root block for interscalene brachial plexus block. **a** The C5 to C8 nerve roots located between the anterior and middle scalene muscles. The C8 nerve root lies on the first rib, and the C5 and C6 nerve roots are fused into the upper trunk of the brachial plexus. **b** Spread of local anesthetic around the C8 nerve root. **c** The separated C5 and C6 nerve roots visualized by the oblique rotation of the medial side of the ultrasonography transducer. **d** Spread of local anesthetic around the C5 and C6 nerve roots

By placing a 5 to 13 MHz linear phased array transducer (UST-5411, Hitachi Aloka Medical, Ltd.) on the supraclavicular fossa, the compact arrangement of the brachial plexus can be visualized lateral to the subclavian artery. The transducer is tracked cephalad to visualize the C8 nerve root lying on the first rib, the C7 nerve root or middle trunk, and the C5 and C6 nerve roots or upper trunk between the anterior and middle scalene muscles in the absence of a visible subclavian artery (Fig. 3a). A 50-mm, 22-gauge nerve-stimulating needle (SonoPlex STIM, Pajunk® GmbH, Geisingen, Germany) passing a current of 0.2 mA at a frequency of 1 Hz, which is generated from a peripheral nerve stimulator (Stimuplex® HNS 12, B. Braun, Melsungen, Germany), is introduced from lateral to medial toward the most caudal cervical nerve root intended to be blocked according to group assignment. The cervical nerve roots are blocked in order from the C7 to C5 nerve roots in group C5–7 and from the C8 to C5 nerve roots in group C5–8 (Fig. 3b). If the upper trunk is visualized, the transducer is rotated with the medial side cephalad to visualize the individual C5 and C6 nerve roots (Fig. 3c). Without performing a new puncture, the trajectory of the needle is adjusted parallel to the ultrasound beam, and each nerve root is blocked (Fig. 3d). An even distribution of 0.75% ropivacaine around each nerve root is achieved by repositioning the needle. Although the plan is to use 25 ml of 0.75% ropivacaine, an additional injection of 5 ml is allowed if there is insufficient spread of the local anesthetic around the cervical nerve roots. If significant resistance against injection of a local anesthetic is encountered [18] or if patients complain of pain or paresthesia, then an intrafascicular injection will be suspected, necessitating the cessation of the injection and subsequent withdrawal and redirection of the needle.

Patients are placed in the sitting position 30 min after the end of local anesthetic injection. Following surgical draping, the bony landmarks and potential portals are outlined carefully. If a pinch test at the skin site 1.5 cm to 3 cm inferior and medial to the posterolateral tip of the acromion induces any pain, 10 ml of 1% lidocaine is infiltrated locally. A surgical incision is made at the same skin site for a posterior portal. A cannula and blunt trocar are inserted anteriorly and medially toward the coracoid process and are passed between the infraspinatus and teres minor muscles. Once the arthroscopic viewing cannula is introduced into the joint capsule, the glenohumeral joint is examined, and subsequent portals are placed as appropriate. At the conclusion of surgery, an indwelling catheter is placed in the subacromial space.

In the sitting position, patients receive oxygen at a rate of 2 L/min via a nasal cannula. Arterial blood pressure is monitored noninvasively at 5-min intervals. More frequent measurements are performed at the discretion of an attending anesthesiologist who is not involved in this study. If the patient complains of pain during surgery, fentanyl is administered in 50 μg increments. If arterial systolic blood pressure ≥170 mmHg or if visualization of the surgical field is impaired despite arterial systolic blood pressure <170 mmHg, nicardipine is administered in 0.25 mg increments. A hypotensive bradycardic event, which is defined as intraoperative bradycardia (a decrease in heart rate of more than 30 beats per minutes [bpm] in less than 5 min compared to the baseline heart rate or any decrease in heart rate to less than 50 bpm at any time) or hypotension (a decrease in arterial systolic blood pressure of more than 30 mmHg in less than 5 min compared to the baseline arterial systolic blood pressure or any decrease in arterial systolic blood pressure to less than 90 mmHg at any time) [19], is treated with inotropes, chronotropes, or vasopressors (e.g., ephedrine, epinephrine, and atropine) at the discretion of the attending anesthesiologist.

When patients begin to feel postoperative pain, 10 ml of a solution containing 25 mg of bupivacaine and 10 μg of epinephrine is injected as a bolus into the operation site via the indwelling catheter, which is then removed. Intravenous tramadol (50 mg) is administered at more than 2-h intervals when patients report a numerical pain rating scale (NRS) score of more than 2 (0 represents “no pain” while 10 represents “the worst pain imaginable”). From the afternoon on the first postoperative day, 650 mg of extended-release acetaminophen and 2 mg of immediate-release hydromorphone are administered orally three times a day. From the second postoperative day, the dosage is changed to four times a day.

### Measurements

As the primary outcome, pain intensity during the introduction of a posterior portal is assessed. The secondary outcomes include:

1. the extent of the ipsilateral sensory, motor, hemidiaphragmatic, and stellate ganglion blockade
2. changes in the results of a pulmonary function test
3. incidence of complications related to ISBPB
4. NRS scores:
  - at admission to the postanesthetic care unit
  - at discharge from the postanesthetic care unit
  - between 6 and 12 h after surgery
  - 24 h after surgery
5. the highest NRS score during the 24 h after surgery
6. postoperative hour when pain starts to be felt
7. patients’ satisfaction with surgical anesthesia and postoperative analgesia
8. dose and frequency of intraoperative and postoperative analgesic use
9. incidence of conversion to general anesthesia

The primary and secondary outcomes will be assessed by EK and CHC.

Pain intensity is rated as 0 (no pain), 1 (mild pain), or 2 (severe pain) during surgical incision and subsequent insertion of the blunt trocar and viewing cannula below the acromion. The sensory and motor blockade is evaluated 30 min after ISBPB. As an assessment of the sensory blockade, the response to the application of ice to the dermatomal areas of C5 to T1 of the shoulder [20] is graded as 0 (no cold sensation), 1 (reduced cold sensation), or 2 (normal cold sensation). Likewise, the motor blockade of the radial, ulnar, median, musculocutaneous, and axillary nerves is assessed by rating the muscle contraction forces (thumb abduction [radial nerve], thumb adduction [ulnar nerve], thumb opposition [median nerve], forearm supination [radial nerve], forearm pronation [median nerve], and shoulder abduction [axillary nerve]) on a scale from 0 to 2, where 0 (complete block), 1 (partial block), and 2 (no block).

Ipsilateral hemidiaphragmatic excursion, bilateral pupil sizes, and pulmonary function are measured in a supine position before and 30 min after the placement of ISBPB. By placing a 1.8 to 5.7 MHz convex phased array transducer (UST-9130, Hitachi Aloka Medical, Ltd.) on the ipsilateral subcostal area, the hyperechoic line of the diaphragm, which moves with the respiratory cycle, is visualized using the liver or spleen as an acoustic window. By tracing the movement of the diaphragm under real-time M-mode ultrasonography, the hemidiaphragmatic excursion is measured as the peak amplitude caused by a deep and quiet inspiration from the resting expiratory position [21]. Hemidiaphragmatic paresis and paralysis are defined as a reduction in hemidiaphragmatic excursion by 25% to 75% and by more than 75% from the baseline, respectively [22].

The stellate ganglion block is confirmed by the development of Horner’s syndrome. As Horner’s syndrome is defined as an ipsilateral pupil diameter that is less than the contralateral pupil diameter by more than 5 mm [23], we measure bilateral pupil diameters 3 min after patients are adapted to low mesopic conditions [24]. Briefly, patients are instructed to focus on the ceiling of the operating room with the eye not being tested. Patients are asked to keep the head straight and both eyes wide open with minimal blinking during targeting and measurement. An automated monocular infrared pupillometer (VIP™-200 pupillometer, NeurOptics Inc., Irvine, CA, USA) is positioned at a right angle to each patient’s axis of vision, with any tilting of the instrument minimized. Average pupil diameter and standard deviation are calculated from the pupillary data sampled at 60 frames per 2 s. The eye contralateral to the site of ISBPB is tested first and then the other eye is tested.

Changes in pulmonary function are determined by changes in the percentage of the predictive values of the measured parameters based on the equations from the European Community for Coal and Steel [25]. Forced expired volume in 1 s (FEV1), forced vital capacity (FVC), FEV1/FVC, and peak expiratory flow rate are measured using a portable spirometer (Micro I, CareFusion, Basingstoke, Hants, United Kingdom). According to the 2005 guidelines of the American Thoracic Society and European Respiratory Society [26], patients inhale completely and rapidly with a pause <1 s at total lung capacity. With a mouthpiece in the mouth and with the lips closed around the mouthpiece, patients exhale maximally until no more air can be expelled. This maneuver is repeated three times, and the best result is taken.

Complications related with ISBPB, which will be recorded prospectively, include accidental puncture of the common carotid, subclavian, dorsal scapular, or vertebral arteries, pneumothorax, hypotensive bradycardic event, epidural and intrathecal injections, local anesthetic systemic toxicity, and neurologic complications. Patients’ satisfaction with the surgical anesthesia and postoperative analgesia is measured using a five-point Likert scale consisting of “very dissatisfied,” “dissatisfied,” “unsure,” “satisfied,” and “very satisfied”.

### Sample size

The primary outcome of this trial is pain intensity during the introduction of a posterior portal. In a pilot study using five subjects for each group, pain intensity was 0 in one patient from group C5–7 and three patients from group C5–8. A pain intensity of 1 was reported by four patients from group C5–7 and two patients from group C5–8. No patient reported a pain intensity of 2. Based on the above results, alternative multinomial distributions (percentage of pain intensity of 0, percentage of pain intensity of 1, percentage of pain intensity of 2) were set as (20%, 80%, 0%) and (60%, 40%, 0%). Using 3000 Monte Carlo samples from the null multinomial distributions (33.3%, 33.3%, 33.3%) and (33.3%, 33.3%, 33.3%) and the alternative distributions, the sample size for this study was calculated to achieve a statistical power of 90% to detect a 0.4 difference in mean pain intensity between the two groups at a significance level of 0.05 using a two-sided Mann–Whitney–Wilcoxon test. Considering a dropout rate of 10%, 37 subjects are required in each group. PASS (version 15.0.3, NCSS, LLC. Kaysville, Utah, USA, ncss.com/software/pass) was used to estimate the sample size.

### Statistical analysis

The data will be analyzed in an intention-to-treat manner, and missing values will be imputed with the last observation carried forward for longitudinal variables, median of the non-missing values for non-longitudinal continuous variables, and mode of the non-missing values for categorical variables. The normality of continuous data will be determined by a Kolmogorov–Smirnov test. Normally and non-normally distributed data will be presented as the mean with standard deviation and median with first to third quartiles, respectively. Ordinal data will also be presented as a median with first to third quartiles. Categorical data will be presented as the number of patients and the percentage [27].

The primary outcome (pain intensity during the introduction of a posterior portal) will be compared between the two groups with a Mann–Whitney–Wilcoxon test. To compare the secondary outcomes between the two groups, independent-samples Student’s *t*-tests will be used for normally distributed continuous data, while Mann–Whitney–Wilcoxon tests will be used for non-normally distributed or ordinal data. Categorical data will be compared between the two groups using Pearson’s chi-square test. Paired samples Student’s *t*-tests or Wilcoxon-signed rank tests will be performed to compare normally or non-normally distributed data that are collected before and 30 min after ISBPB. Generalized estimating equations will be used to model changes in NRS scores over time and subsequently to investigate between-subject, within-subject, and interaction effects. All statistical tests are two-sided, and a level of significance <0.05 is considered statistically significant. The statistical analysis will be performed using IBM SPSS Statistics software (version 25, IBM Corp., Armonk, NY, USA) by a statistician who is not involved in data collection.

## Discussion

The two main side effects of ISBPB, which are ipsilateral hemidiaphragmatic paresis or paralysis and stellate ganglion blockade, do not have clinical significance because their symptoms, such as dyspnea, ptosis, facial flushing, or nasal stuffiness, are usually not noticed and do not cause significant discomfort in healthy patients [2, 28]. However, the insufficient blockade of the lower trunk (C8–T1) by ISBPB [6] results in significant pain and discomfort in the posterior aspect of the shoulder [7] and requires additional infiltration of local anesthetic. Hence, to overcome the limitation of conventional ISBPB (sparing of the lower trunk), a more caudal approach is used [8–11]. Although the caudal approach anesthetizes the ulnar sides of the elbow, forearm, and hand, its effectiveness in anesthetizing the posterior aspect of the shoulder has not been evaluated [8–11].

Of the two major dermatome maps (Keegan and Garrett’s [20] and Foerster’s [29]), which are widely cited in standard anatomy textbooks [30], we chose Keegan and Garrett’s map to assess the sensory blockade produced by ISBPB because its dermatomes correlate very well with the range of the sensory blockade produced by ISBPB, which consistently spares the posterior aspect of the shoulder [7]. According to Keegan and Garrett’s dermatome map, the posterior aspect of the shoulder is innervated by the C8 nerve root, which is not blocked by ISBPB [10, 11]. However, Foerster’s dermatome map shows the innervation of the posterior shoulder by the C5 nerve root, which is routinely blocked during ISBPB. Therefore, the use of Keegan and Garrett’s map is justified in this trial.

Regrettably, previous studies did not consider the C8 nerve root block during ISBPB even after the universal utilization of ultrasound guidance [10, 11], which ensures the accurate placement of a local anesthetic around nerve roots. Compared to the C5, C6, and C7 nerve roots, the C8 nerve root is more difficult to visualize under ultrasonography [14–16]. However, the C8 nerve root block is worth trying because the probability of its visualization has been reported to be as high as 80% [16]. When the C8 nerve root is visualized on the first rib, the C5 and C6 nerve roots mostly coalesce into the upper trunk [31]. To obtain a view of the individual C5 and C6 nerve roots, we obliquely rotate the medial side of the ultrasonography transducer cephalad, which does not require a new skin puncture. In this way, local anesthetic can be placed around each nerve root.

In this study, we chose pain intensity as the primary outcome during the incision and insertion of an arthroscopic port on the posterior aspect of the shoulder rather than the sensory blockade corresponding to each nerve root due to the individual variation in the distribution of dermatomes on the posterior aspect of the shoulder [30, 32]. Additionally, we measure the incidence of the hemidiaphragmatic block and Horner’s syndrome, which have not been completely averted to date. To determine the exact incidence, we use a multi-modality approach with ultrasonography, spirometry, and a digital pupillometer. This is the first use of a digital pupillometer to measure the change in pupil size following ISBPB. With this equipment, we expect to determine the quantitative differences between the two techniques.

This is the first randomized controlled trial to investigate whether a C8 block during ISBPB is effective in reducing pain intensity upon an insertion of a posterior portal into the posterior aspect of the shoulder. It is expected that any positive results produced by this new technique will improve the quality of surgical anesthesia provided by ISBPB for patients undergoing arthroscopic shoulder surgery.

### Trial status

Following the approval of the study protocol (CR-18-018), the recruitment of subjects commenced in April 2018 and was completed in August 2019.

## Additional file

Additional file 1: SPIRIT checklist. (DOC 122 kb)

## Data Availability

The datasets used or analyzed during the current study will be available from the corresponding author on reasonable request.

## Abbreviations

bpm: beats per minute
CONSORT: Consolidated Standards of Reporting Trials
FEV1: Forced expired volume in one second
FVC: Forced vital capacity
ISBPB: Interscalene brachial plexus block
NRS: Numerical pain rating scale
SPIRIT: Standard Protocol Items: Recommendations for Interventional Trials
